# A Rare Case of Ectopic Bile Duct in the Third Portion of the Duodenum

**DOI:** 10.7759/cureus.107489

**Published:** 2026-04-21

**Authors:** Hassan Ahmed Daoud, Youssef Sakhy, Oumaima Iraqui Houssaini, Abdellatif Siwane, Omar Kacimi

**Affiliations:** 1 Emergency Department of Radiology, Ibn Rochd University Hospital Center, Faculty of Medicine and Pharmacy of Casablanca, Hassan II University, Casablanca, MAR

**Keywords:** common bile duct, duodenum, ectopia, mrcp, rare case report

## Abstract

Ectopic drainage of the common bile duct into the third portion of the duodenum is an extremely rare anatomical anomaly of the biliary tract, often overlooked due to its asymptomatic nature. Its diagnosis relies primarily on imaging, particularly MR cholangiography, which allows for accurate and non-invasive analysis of the biliary tree. Recognition of this variant is important, as it can lead to atypical clinical presentations and pose diagnostic and therapeutic challenges, particularly due to potential difficulties during endoscopic procedures such as endoscopic retrograde cholangiopancreatography (ERCP), linked to the unusual location of the biliary orifice.

Here, we report a case of a patient with a prior cholecystectomy who presented with diffuse abdominal pain, without fever or abnormal laboratory findings. Initial ultrasound was normal, and MR cholangiography revealed an abnormal common bile duct orifice in the third portion of the duodenum. This case illustrates the value of imaging in identifying this rare variant and highlights the importance of its recognition, particularly to anticipate potential technical difficulties during endoscopic procedures.

## Introduction

Anatomical variations of the biliary tract are common, but some remain exceptionally rare and poorly understood. Among these, ectopic opening of the common bile duct into an unusual part of the digestive tract constitutes a congenital developmental anomaly that has been reported only rarely in the literature. The majority of cases described involve drainage into the stomach or the proximal duodenum, particularly at the level of the bulb [1,2]. In contrast, a junction in the third portion of the duodenum (D3) is extremely unusual [3].

These biliary variants are most often asymptomatic and discovered incidentally during imaging examinations. However, they can sometimes cause a variety of clinical symptoms, such as abdominal pain, abnormal liver function tests, or repeated failures of endoscopic retrograde cholangiopancreatography (ERCP), due to the unusual location of the papilla [4]. Advances in imaging techniques, particularly MR cholangiography, now allow for the precise, non-invasive, and essential characterization of these anatomical anomalies. Recognizing these variants is crucial to avoid diagnostic errors and to adapt the therapeutic strategy, particularly endoscopic treatment [5]. We report here a rare case of ectopic opening of the common bile duct into the third portion of the duodenum, confirmed by imaging, and provide a brief review of the literature to highlight the diagnostic and clinical implications.

## Case presentation

A 47-year-old female patient, with a history of cholecystectomy in 2020, presented with diffuse, non-specific abdominal pain for several days, without fever, jaundice, or bilious vomiting. Physical examination revealed a soft, non-distended abdomen with moderate tenderness in the epigastrium and right hypochondrium. Initial laboratory tests, including transaminases, gamma-glutamyltransferase, total and conjugated bilirubin, and alkaline phosphatase, were within normal limits. The patient was referred to us for an abdominal ultrasound, which showed a liver of normal appearance, with no dilation of the intra- or extrahepatic bile ducts and an empty gallbladder.

**Figure 1:**
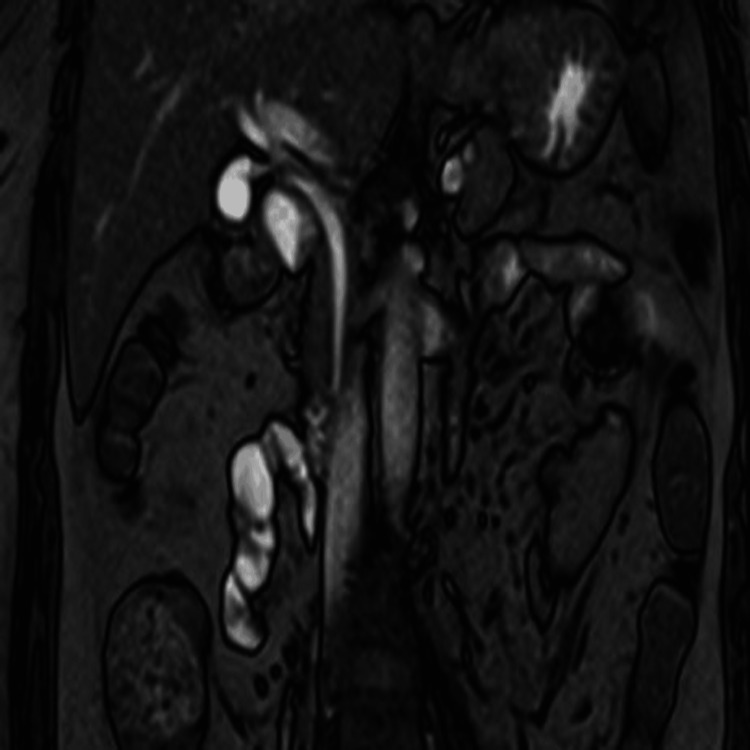
Coronal T2 TrueFISP. Image depicting an ectopic insertion of the common bile duct into the third portion of the duodenum (D3). TrueFISP: true fast imaging with steady-state precession

**Figure 2:**
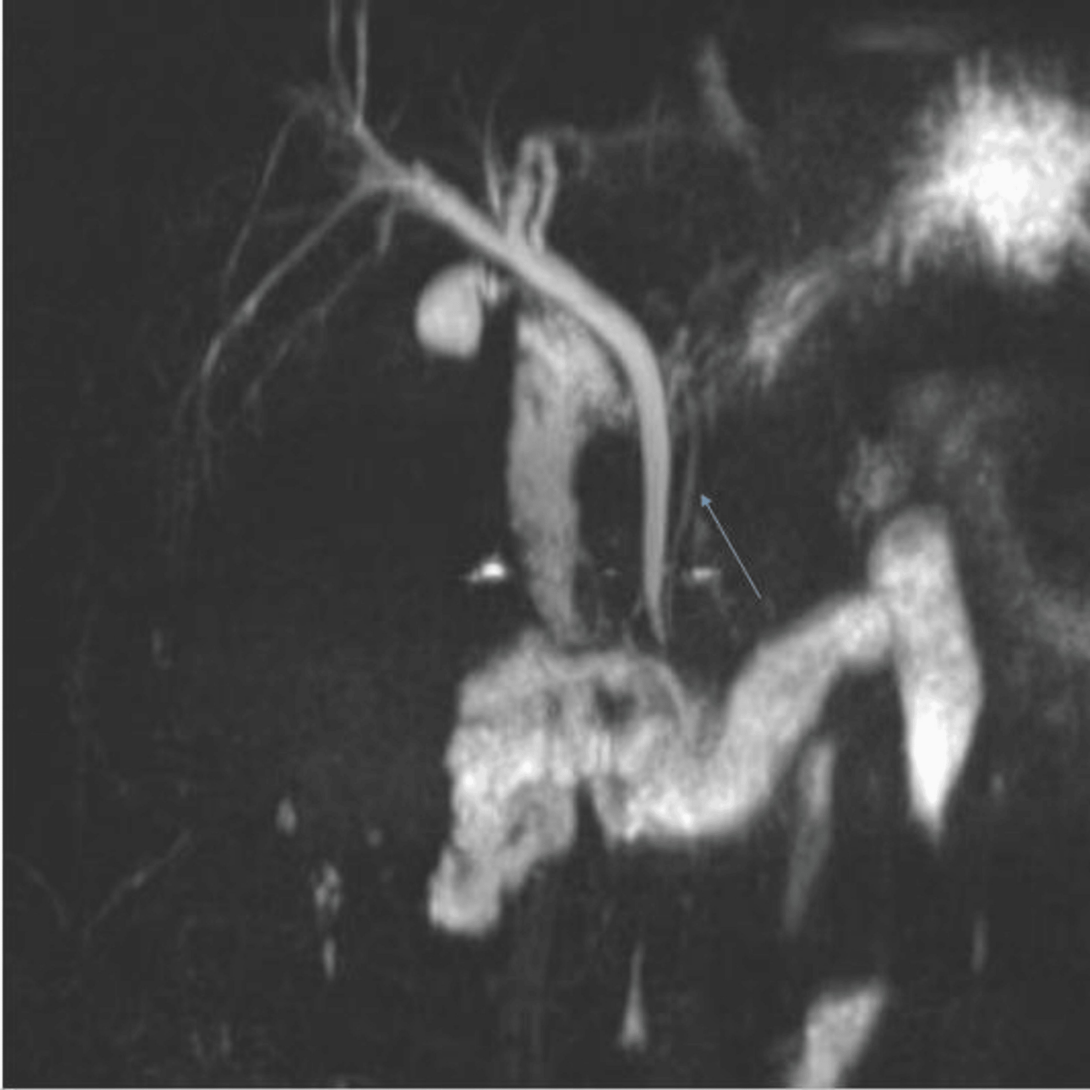
Coronal heavily T2-weighted MR cholangiopancreatography (MRCP). Image demonstrating a low insertion of the common bile duct, draining into the third portion of the duodenum with the pancreatic duct (arrow) seen to converge with the common bile duct upstream of the ectopic orifice.

As symptoms persisted, an MR cholangiography was performed. This incidentally revealed an ectopic opening of the common bile duct into the third portion of the duodenum (D3) (Figures 1, 2). The common bile duct followed a continuous distal course before its ectopic insertion, with a normal diameter and no evidence of stones, stenosis, or morphological abnormalities. The main pancreatic duct was seen to converge with the common bile duct upstream of the ectopic orifice, suggesting a common channel. No normal papillary opening was identified in the expected location within the second portion of the duodenum. Furthermore, no pancreatic or duodenal mass was detected. The patient was referred back to her general practitioner for follow-up, with no indication for immediate intervention. Clinical monitoring was recommended.

## Discussion

Ectopic opening of the common bile duct into the third portion of the duodenum (D3) is an extremely rare anatomical variant, markedly distinct from more common ectopic locations such as the duodenal bulb or the stomach [1,2]. In embryology, the hepatic diverticulum divides into the following two parts: the pars hepatica, which gives rise to the hepatic ducts, and the pars cystica, which forms the gallbladder and cystic duct. The common bile duct develops from the shared portion during embryogenesis. Early separation of the hepatic diverticulum may result in incomplete fusion, leading to the formation of duplicated bile ducts. The site of biliary drainage depends on the timing of development. If an accessory bile duct forms before the separation of the stomach and duodenum, it may open into the stomach; if it develops after this separation, it typically drains into the duodenum. Normally, the fusion of the bile and pancreatic ducts creates the common duct, which opens into D2. An opening into D3 implies a failure of regression or distal migration of the fusion point [1].

Clinically, this variant is often asymptomatic and discovered incidentally. When symptoms are present, they are generally non-specific - abdominal pain, dyspepsia, subtle abnormalities in liver function tests, or episodes of pancreatitis in some cases. However, cases of gallstones, cholangitis, or pancreatitis have been reported in the literature, particularly when associated with stenosis or an unusual duct length [6].

In terms of imaging, MR cholangiography has established itself as the modality of choice for non-invasive anatomical characterization, enabling complete mapping of the biliary tree without the use of contrast agents and clearly identifying the absence of a normal D2 junction [5,7]. In our case, this allowed a clear demonstration of ectopic drainage at D3, without dilation or obstruction.

Recognizing this anatomical variant is essential for appropriate endoscopic management. The absence of the papilla at its usual location in the second portion of the duodenum (D2) may lead to failure of ERCP or difficulty in biliary cannulation. A thorough understanding of biliary anatomical variations is therefore crucial for both radiologists and endoscopists to anticipate these challenges and adapt the procedure accordingly [8].

## Conclusions

Ectopic opening of the common bile duct into the third portion of the duodenum is a rare anatomical anomaly, often asymptomatic and discovered incidentally. MR cholangiography is the key diagnostic tool, enabling precise and non-invasive characterization of this variant. Recognition of this anomaly is essential to avoid diagnostic errors and prevent failures of endoscopic procedures such as ERCP. This case illustrates the importance of the radiologist’s role in identifying this rare variant to ensure optimal management.
